# Association of Adiposity and Mental Health Functioning across the Lifespan: Findings from *Understanding Society* (The UK Household Longitudinal Study)

**DOI:** 10.1371/journal.pone.0148561

**Published:** 2016-02-05

**Authors:** Apostolos Davillas, Michaela Benzeval, Meena Kumari

**Affiliations:** 1 Institute for Social and Economic Research (ISER), University of Essex, Colchester, United Kingdom; 2 MRC/CSO Social and Public Health Sciences Unit, University of Glasgow, Glasgow, United Kingdom; 3 Department of Epidemiology and Public Health, University College London, London, United Kingdom; McMaster University, CANADA

## Abstract

**Background:**

Evidence on the adiposity-mental health associations is mixed, with studies finding positive, negative or no associations, and less is known about how these associations may vary by age.

**Objective:**

To examine the association of adiposity -body mass index (BMI), waist circumference (WC) and percentage body fat (BF%)- with mental health functioning across the adult lifespan.

**Methods:**

Data from 11,257 participants (aged 18+) of *Understanding Society*: the UK Household Longitudinal Study (waves 2 and 3, 5/2010-7/2013) were employed. Regressions of mental health functioning, assessed by the Mental Component Summary (MCS-12) and the General Health Questionnaire (GHQ-12), on adiposity measures (continuous or dichotomous indicators) were estimated adjusted for covariates. Polynomial age-adiposity interactions were estimated.

**Results:**

Higher adiposity was associated with poorer mental health functioning. This emerged in the 30s, increased up to mid-40s (all central adiposity and obesity-BF% measures) or early 50s (all BMI measures) and then decreased with age. Underlying physical health generally accounted for these associations except for central adiposity, where associations remained statistically significant from the mid-30s to50s. Cardiovascular, followed by arthritis and endocrine, conditions played the greatest role in attenuating the associations under investigation.

**Conclusions:**

We found strong age-specific patterns in the adiposity-mental health functioning association that varied across adiposity measures. Underlying physical health had the dominant role in attenuating these associations. Policy makers and health professionals should target increased adiposity, mainly central adiposity, as it is a risk factor for poor mental health functioning in those aged between mid-30s to 50 years.

## Introduction

Mental health is recognized as a global public health concern [1]. Understanding the predictors of mental health throughout the lifespan may help to elucidate pathways that promote mental health and prevent mental disorders. One hypothesized predictor is adiposity [2].

However, evidence of the association of adiposity with different measures of mental health, such as depressive symptoms and mental health-related quality of life (MHRQoL), is equivocal. While some studies support the “fat and sad” hypothesis (higher weight is associated lower mental health) [3–5], this is far from a universal finding [6–8]. A systematic review described positive associations of adiposity with depressive symptoms in the United States (US) for women but failed to find evidence in non-US cross-sectional studies [9], which the authors attributed to possible unique cultural disparities between the US and other populations.

Inconsistencies in these studies may also relate to both gender and age differences in associations. Previous evidence suggests that the association between adiposity and mental health functioning may be limited to women, with findings more mixed for men [10, 11], for whom there is some support for the “jolly fat” hypothesis, i.e. higher weight being associated with better mental health [12, 13]. A recent meta-analysis suggested that obese women experienced lower MHRQoL compared to their healthy weight counterparts, whereas the opposite was the case for obese men [14]. On the other hand, another meta-analysis did not find gender-specific differences, and suggested that overweight people had better MHRQoL while those severely obese experienced significantly reduced MHRQoL [15].

Although age may be a potential moderator of the association between adiposity and mental health [2, 10, 16], it has received limited attention in the literature, being explored in very few studies, without a consistent pattern emerging. For example, Larson et al. found that obese Swedish women aged 35–64, but not younger, experienced lower MHRQoL [16]. A US study showed that higher BMI was associated with depressive mood among those aged 18–65 but not in older age groups [11].

Many of the reported studies are limited to unadjusted/poorly adjusted associations or fail to account for underlying chronic diseases [3–5, 9]. Controlling for underlying physical health may be important as common chronic conditions such as arthritis and cardio-metabolic conditions are associated with both obesity [17, 18] and mental health [19–21]. Moreover, many existing studies rely on BMI (measured or self-report) and most of them are limited in their assessment of alternative measures of adiposity [3, 8–10, 15]. Different measures of adiposity may shed light on the underlying biological mechanisms [22]. For example, it is suggested that central, rather than general, adiposity may be more salient to depressive symptoms [23].

In this study we examine the association of adiposity—measured by BMI, waist circumference (WC) and percentage body fat (BF%)—with mental health functioning, assessed by both psychological distress and MHRQoL, using a large national representative sample (*Understanding Society*: the United Kingdom Household Longitudinal Study; UKHLS). We capitalize on the wide age range available to investigate how the adiposity-mental health functioning association varies by age, allowing for flexible associations between age, adiposity and mental health functioning. Finally, we explore the robustness of these associations after accounting for socio-economic factors, smoking and physical health.

## Methods

### Sample

The data were from the General Population Sample (GPS) of the UKHLS, which is a stratified clustered random sample of households representative of the United Kingdom population [24, 25]. Five waves of data are currently available; this paper focuses on respondents who had a nurse visit as part of wave 2 and were followed up in wave 3. UKHLS is available through the UK Data Archive under the end-user license [26, 27].

At wave 1 (1/2009-3/2011) the GPS had a response rate of 57.3% at the household level; the individual response rate was 87% within the co-operating households (26,057 households; 43,674 individuals aged 16+). Response rates at waves 2 (1/2010-3/2012) and 3 (1/2011-7/2013) were around 80% for households and 90% for individuals [24].

The nurse visit took place after the wave 2 main interview [28]; respondents were eligible if they took part in the main interview, were aged 16+, lived in Great Britain (not Northern Ireland), conducted their interview in English and were not pregnant (26,699 individuals). Of these 15,647 took part (response rate of 58.6%), and 13,135 of those were followed up in wave 3 and completed any part of the self-completion instruments (which included the measures of mental health functioning). Restricting our analysis to individuals aged 18+ to focus on adult obesity [29], resulted in a potential sample of 12,967 adults.

Participants gave informed oral consent to take part in each wave of the study (written consent was obtained for blood sample collection). Participants were enrolled only after consent was provided. The UKHLS has been approved by the University of Essex Ethics Committee and the nurse data collection by the National Research Ethics Service.

### Measures

#### Mental Health Functioning Outcomes

Two measures were employed: the 12-item General Health Questionnaire (GHQ-12) and the Mental Component Summary score (MCS-12), derived from the 12-item Short Form Health Survey (SF-12). Both measures were obtained by computer based self-completion instruments at wave 3. The GHQ-12 is a widely used measure of non-psychotic psychological distress [30] with excellent psychometric properties [30, 31]. The Likert scoring method, ranging from 0 (least distressed) to 36 (most distressed), was applied. This approximates to a normal distribution and can be treated as a continuous measure in multivariate analysis [32].

The MCS-12 is a well-established summary measure of MHRQoL [33]. The scores are standardized to US population norms with a mean score set at 50 (standard deviation = 10); higher scores indicate better mental health functioning. Compared to the GHQ-12, which aims to measure psychological distress and the associated psycho-social dysfunction, the MCS-12 is a measure of quality of life related to the broader context of mental health [34]. To facilitate consistency of interpretation between GHQ-12 and MCS-12 scores, the GHQ-12 was inverted such as higher values indicate better mental health functioning. The inverted GHQ-12 will be referred to as GHQ-12 for ease of reference.

#### Measures of Adiposity

All the adiposity measures were taken during the wave 2 nurse visits [28]. Respondents were required to remove their socks, shoes and any bulky clothing. Height was measured using a portable stadiometer, with the head in the Frankfort Plane. Body weight and BF% (using the bioelectrical impedance analysis) were measured using a portable digital floor body fat monitor/scale (Tanita BF 522). BMI is defined as weight (kilograms) over the square of height (meters). WC was measured at the midpoint between the lower rib and the upper margin of the iliac crest. The measurement, recorded to the nearest millimeter, was taken twice or with a third measurement if the two measurements differ by >3cm. The mean of the two valid measurements (the two closest if there were three) were used in the analysis.

Dichotomous obesity measures were also examined. For BMI, people with BMI≥30 kg/m^2^ were classified as obese [35]. For BF% obesity was defined using Gallagher’s equivalent BF% charts [36], which are gender, age and ethnicity specific ranges that link BMI categories to BF%. Abdominal obesity was defined as WC≥102cm for males and as WC≥88cm for females [35].

#### Covariates

A large set of covariates was included in the analysis; most were collected at wave 3 unless otherwise indicated. Demographic characteristics included age, gender and ethnicity (white, mixed, Asian and Black). Socio-economic characteristics included educational attainment (university degree, other higher degree, a-level, o-level/basic qualifications or no-qualifications) and equivalised (using the Organization for Economic Co-operation and Development (OECD) modified scale) household gross income [28]. Marital status (single, married/cohabitated, divorced/separated or widowed) and smoking behaviour (current, ex-smoker or never smoker; based on wave 2 data) were also accounted for in the analyses.

Current chronic health conditions were based on self-report of doctor diagnosis of specific health conditions across waves 1 to 3 (questions were asked initially at wave 1, and repeated for newly diagnosed conditions at waves 2 and 3). These were grouped as: respiratory diseases (asthma, emphysema and chronic bronchitis), cardiovascular diseases (congestive heart failure, coronary heart disease, angina, heart attack, stroke and hypertension), endocrine diseases (hyperthyroidism, hypothyroidism and diabetes mellitus), arthritis and other diseases (liver conditions, cancer and epilepsy). We also included dummies for region (9 regions of England, Wales and Scotland) and urbanization indicators (urban or rural residence) as covariates. Finally, the number of days between wave 3 and nurse visits at wave 2 was captured using a time gap variable.

### Data Analysis

The analyses accounted for the complex survey design, adjusting for clustering and stratification [37], using the svy commands in Stata 13 [38]. Probability weights were employed to account for non-response and unequal selection at wave 1 as well as eligibility and attrition at subsequent waves. We calculated these by adjusting published weights for the wave 2 nurse visit [28], to account for non-response at wave 3 main survey and the self-complete instruments, which was estimated by backward stepwise logistic regressions on predictors from the nurse visit.

Linear regressions were estimated to explore the association between adiposity and mental health functioning. Our analyses were conducted on the pooled sample of men and women since gender-adiposity interactions were not significant at the 5% level. Separate regression models were run for each adiposity measure. In addition to employing continuous measures of adiposity, we also explored the presence of non-linearities using discrete obesity measures. Preliminary analysis including the full set of adiposity categories for BMI (underweight, <18.5 kg/m^2^; normal weight, 18.5–24.9 kg/m^2^; overweight 25–29.9 kg/m^2^ and obese), WC (normal-WC, <94 or <80cm for males and females; moderate-WC, 94–101.9 or 80–87.9cm, and abdominal obese) and BF% (four categories following Gallagher’s charts) [35, 36, 39], showed that coefficients for lower and moderate adiposity categories did not differ from each other (at the 10% level); therefore the former were grouped together. The base model included demographic, regional variables and the time gap between wave 3 and nurse visits. A cubic polynomial in age was included as this provided a better statistical fit compared to linear, quadratic and quartic associations for both mental health measures.

Heterogeneity in the adiposity-mental health functioning association by age was tested using interaction terms between the polynomial age variables and the adiposity measures. In cases where non-significant (at the 5% level) cubic interactions were observed (i.e., the mental health-BMI models), linear and quadratic interactions were included. Results are presented graphically to illustrate the associations at different ages effectively. These were obtained from linear combinations of the main adiposity coefficients and the age-adiposity interactions across different ages; the margins command in Stata 13 was used to calculate these associations and the 95% confidence intervals (CI). For the continuous adiposity measures, the graphs show the predicted average difference in the mental health functioning scores for a 1-unit difference in the adiposity measures, across different ages, holding the remaining covariates constant. For the dichotomous obesity measures, predicted differences in mental health functioning between obese/non-obese by age are presented. Centring the age variable resulted into identical adiposity gradients [40]; thus, the raw age values were utilized.

Robustness of these associations was tested by adjusting the base models with socio-economic characteristics, marital status and smoking behaviour (“without health” models). In subsequent analysis, all physical health conditions were simultaneously added to the models (“full models”) in order to explore their joint role on influencing the observed associations across different ages. Physical conditions were also separately added to the “without health” models to explore the individual role of each of the physical conditions. In all the models accounting for physical health, interactions of health conditions with age were tested and included when significant at the 5% level.

## Results

From the potential sample of 12,967 individuals, 36 had missing mental health functioning information, 1,035 had no adiposity measures and 639 lacked data on the remaining covariates (588 of them due to missing information on health conditions), giving an analytical sample of 11,257 individuals. Comparing this analytical sample with the potential sample, average mental health functioning scores, average BMI and BF% were very similar whereas mean WC was somewhat lower in the analytical sample (92.9 vs 93.4 cm). Moreover, people in our analytical sample were somewhat younger (47.8 vs 48.5 years) and slightly less likely to suffer from arthritis (prevalence: 15.9 vs 16.8%), cardiovascular (19.7 vs 21.3%) and endocrine (9.06 vs 9.88%) diseases. The remaining variables were identical across both samples.

### Sample Characteristics

Table 1 shows the characteristics of the analytical sample by age groups, which were selected to illustrate differences at different life stages. MCS-12 and GHQ-12 scores varied across age groups with the highest mean values in the 65–74 age group. Mean values for the continuous adiposity measures and obesity prevalence increased non-monotonically by age. On average, people under 40 years had higher education levels and were more likely to be current smokers than those in older age groups. Lower income was experienced by those aged over 64 compared to those younger. Variations in the marital status were also observed by age, with the proportion married/cohabiting increasing until the age of 75 years. As expected, prevalence of most health conditions was higher among older respondents. Statistically significant age trends were evident in all the aforementioned variables (p-values<0.001).

**Table 1:** Participant characteristics for the analysis sample of UKHLS (waves 2–3, 5/2010-7/2013) by age group^a^.

	Age group					
	18–29 (n = 1,189)	30–39 (n = 1,743)	40–64 (n = 5,574)	65–74 (n = 1,817)	75+ (n = 934)	Total sample (n = 11,257)
**Continuous variables**	**Mean [sd]**	**Mean [sd]**	**Mean [sd]**	**Mean [sd]**	**Mean [sd]**	**Mean [sd]**
MCS-12	47.32 [10.03]	48.11 [9.61]	49.39 [9.59]	52.75 [8.41]	52.29 [9.36]	49.42 [9.69]
GHQ-12 Likert (inverted)	24.93 [5.63]	24.83 [5.57]	24.67 [5.62]	26.14 [4.29]	26.06 [4.39]	25.03 [5.41]
BMI (kg/m^2^)	25.42 [5.05]	27.10 [5.13]	28.35 [5.28]	28.65 [4.77]	27.91 [4.41]	27.59 [5.22]
BF%	25.47 [11.34]	28.80 [10.81]	31.22 [10.77]	32.84 [10.49]	31.46 [11.07]	29.95 [11.13]
WC (cm)	84.91 [13.05]	90.25 [13.22]	95.22 [13.92]	97.97 [13.23]	96.40 [12.43]	92.88 [14.15]
Age (years)						47.75 [17.7]
HH income (monthly, thousand £)	1.94 [1.21]	2.21 [1.37]	2.30 [1.64]	1.75 [1.25]	1.53 [1.64]	2.09 [1.5]
**Categorical variables**	**% [95% CI]**	**% [95% CI]**	**% [95% CI]**	**% [95% CI]**	**% [95% CI]**	**% [95% CI]**
*BMI classification*						
Obese	17.55 [14.9; 20.2]	23.05 [20.7; 25.4]	33.00 [31.7; 34.4]	35.28 [32.9; 37.7]	27.94 [24.6; 31.3]	28.29 [27.2; 29.3]
Non-obese	82.45 [79.8; 85.1]	76.95 [74.6; 79.3]	67.00 [65.6; 68.3]	64.72 [62.3; 67.1]	72.06 [68.7; 75.4]	71.71 [70.7; 72.8]
*BF% classification*						
Obese	21.07 [18.4; 23.8]	30.01 [27.3; 32.7]	33.11 [31.6; 34.7]	32.75 [30.3; 35.2]	26.78 [23.6; 30.0]	29.79 [28.7; 30.9]
Non-obese	78.93 [76.3; 81.6]	69.99 [67.3; 72.7]	66.89 [65.3; 68.4]	67.25 [64.8; 69.7]	73.22 [70.0; 76.4]	70.21 [69.1; 71.3]
*WC classification*						
Abdominal obese	21.26 [18.5; 24.0]	30.19 [27.7; 32.7]	46.88 [45.3; 48.5]	56.96 [54.5; 59.4]	54.62 [50.8; 58.4]	41.07 [39.9; 42.2]
Non-abdominal obese	78.74 [76.0; 81.5]	69.81 [67.3; 72.3]	53.12 [51.5; 54.7]	43.04 [40.6; 45.5]	45.38 [41.6; 49.2]	58.93 [57.8; 60.1]
*Gender*						
Males	47.78 [44.4; 51.1]	47.57 [45.0; 50.1]	47.79 [46.6; 49.0]	47.50 [45.3; 49.7]	45.02 [41.9; 48.2]	47.49 [46.5; 48.4]
Females	52.22 [49.0; 55.6]	52.43 [49.9; 55.0]	52.21 [51.0; 53.4]	52.50 [50.3; 54.5]	54.98 [51.8; 58.1]	52.51 [51.6; 53.5]
*Ethnic groups*						
White	84.44 [81.2; 87.7]	83.53 [80.7; 86.3]	92.02 [90.8; 93.3]	97.00 [95.8; 98.2]	96.04 [93.4; 98.1]	90.06 [88.9; 91.2]
Mixed ethnic groups	2.95 [1.6; 4.3]	1.39 [0.7; 2.0]	0.86 [0.6; 1.2]	0.41 [0.1; 0.7]	0.80 [0.1; 1.5]	1.28 [1.0; 1.6]
Asian/Asia British	8.17 [5.7; 10.6]	10.73 [8.2; 13.2]	4.16 [3.3; 5.0]	1.62 [0.7; 2.5]	1.98 [0.5; 3.5]	5.57 [4.7; 6.5]
Black/Black British	4.44 [2.6; 6.3]	4.36 [2.9; 5.8]	2.96 [2.1; 3.8]	0.97 [0.3; 1.6]	1.18 [0.2; 2.5]	3.09 [2.5; 3.7]
*Educational level*						
University degree	23.33 [20.4; 26.2]	35.05 [32.2; 37.9]	25.89 [24.5; 27.3]	13.90 [12.2; 15.6]	11.58 [9.2; 14.0]	24.42 [23.4; 25.5]
Other higher degree	9.01 [7.1; 10.9]	14.88 [12.6; 17.1]	13.37 [12.4; 14.3]	11.33 [9.9; 12.8]	11.46 [9.4; 13.6]	12.43 [11.7; 13.2]
A-level	38.15 [34.9; 41.4]	21.63 [19.3; 23.9]	17.52 [16.3; 18.7]	13.46 [11.7; 15.2]	9.11 [7.0; 11.3]	20.90 [19.9; 21.9]
O-level/Basic qualifications	25.97 [23.0; 28.9]	24.97 [22.6; 27.3]	32.65 [31.2; 34.1]	33.53 [31.0; 35.9]	28.95 [25.6; 32.4]	29.89 [28.9; 30.9]
No qualification	3.54 [2.2; 4.8]	3.48 [2.3; 4.6]	10.56 [9.6; 11.6]	27.78 [25.2; 30.3]	38.89 [35.1; 42.7]	12.36 [11.6; 13.2]
*Marital status*						
Single	85.10 [82.6; 87.5]	37.09 [34.1; 40.1]	14.43 [13.3; 15.6]	4.25 [3.2; 5.3]	5.90 [4.1; 7.7]	29.54 [28.3; 30.8]
Married/cohabitated	14.08 [11.7; 16.5]	57.05 [53.9; 60.2]	65.88 [64.3; 67.4]	69.76 [67.3; 72.2]	49.20 [45.0; 53.4]	53.90 [28.3; 30.8]
Divorce/separated	0.80 [0.4; 1.3]	5.67[4.4; 7.0]	16.87 [15.8; 18.0]	12.81 [11.1; 14.5]	6.53 [4.7; 8.4]	10.63[10.0; 11.3]
Widowed	0.05 [0.0; 0.2]	0.20 [0.0; 0.5]	2.81 [2.3; 3.3]	13.17 [11.5; 14.9]	38.38 [34.6; 42.2]	5.93 [5.5; 6.4]
Smoking						
Current smoker	26.54 [23.4; 29.6]	25.94 [23.3; 28.6]	23.33 [21.9; 24.8]	12.26 [10.5; 14.0]	6.85 [4.7; 9.0]	21.73 [20.7; 22.8]
Ex-smoker	17.71 [15.4; 20.1]	28.50 [26.0; 31.0]	31.70 [30.3; 33.1]	47.67 [45.3; 50.1]	47.15 [43.6; 50.7]	31.71 [30.7; 32.7]
Never smoker	55.76 [52.5; 59.1]	45.56 [42.6; 48.5]	44.96 [43.4; 46.5]	40.07 [37.8; 42.4]	46.00 [42.2; 49.8]	46.56 [45.4; 47.7]
*Arthritis*						
Presence	1.48 [0.7; 2.2]	4.32 [3.3; 5.4]	16.71 [15.6; 17.8]	35.68 [33.3; 38.1]	40.16 [36.6; 43.7]	15.89 [15.1; 16.7]
Not present	98.52 [97.8; 99.3]	95.68 [94.6; 96.7]	83.29 [82.2; 84.4]	64.32 [61.9; 66.7]	59.84 [56.3; 63.4]	84.11 [83.3; 84.9]
*Respiratory diseases*						
Presence	12.76 [10.7; 14.8]	10.41 [8.9; 11.9]	11.38 [10.5; 12.3]	12.34 [10.6; 14.0]	12.55 [9.9; 15.2]	11.67 [11.0; 12.4]
Not present	87.24 [85.2; 89.3]	89.59 [88.1; 91.1]	88.62 [87.7; 89.5]	87.66 [86.0; 89.4]	87.45 [84.8; 90.1]	88.33 [87.6; 89.0]
*Cardiovascular diseases*						
Presence	2.01 [1.0; 3.0]	4.00 [3.0; 5.0]	19.54 [18.3; 20.7]	44.24 [41.6; 46.9]	59.26 [55.7; 62.8]	19.73 [18.9; 20.6]
Not present	97.99 [97.0; 99.0]	96.00 [95.0; 97.0]	80.46 [79.3; 81.7]	55.76 [53.1; 58.4]	40.74 [37.2; 44.3]	80.27 [79.4; 81.1]
*Endocrine diseases*						
Presence	1.77 [1.1; 2.5]	3.36 [2.5; 4.2]	9.65 [8.8; 10.5]	19.18 [17.2; 21.2]	19.83 [16.9; 22.8]	9.06 [8.5; 9.6]
Not present	98.23 [97.5; 98.9]	96.64 [95.8; 97.5]	90.35 [89.5; 91.2]	80.82 [78.8; 82.8]	80.17 [77.2; 83.1]	90.94 [90.4; 91.5]
*Other disease*						
Presence	1.81 [1.02; 2.6]	1.90 [1.2; 2.6]	3.24 [2.7; 3.8]	5.65 [4.3; 7.0]	6.13 [4.5; 7.8]	3.27 [2.9; 3.6]
Not present	98.19 [97.4; 99.0]	98.10 [97.4; 98.8]	96.76 [96.3; 97.3]	94.35 [93.0; 95.7]	93.87 [92.2; 95.5]	96.73 [96.4; 97.1]

Abbreviations: BF%, percentage body fat; BMI, body mass index; CI, confidence interval; GHQ-12, 12-item General Health Questionnaire; HH, household; MCS-12, Mental Component Summary of the 12-item Short Form Health Survey; n, number of observations; sd; standard deviation; WC, waist circumference.

^a^ Age groups selected to reflect different life stages. Descriptive statistics were adjusted to account for sample weights and complex survey design.

### Association between Adiposity and Mental Health Functioning

Table 2 shows the association of adiposity measures (continuous and dichotomous obesity measures) with mental health functioning adjusted for basic covariates (demographics, regional variation and the follow up time gap variable). Focusing on the continuous adiposity measures, BMI was negatively associated with GHQ-12 (β = -0.046; p-value<0.001) and MCS-12 (β = -0.055; p-value: 0.011), indicating poorer mental health functioning was correlated with higher BMI. For example, holding all the other covariates constant, a higher BMI by two standard deviations (s.d.), i.e. 10.4 kg/m^2^, was associated with a lower GHQ-12 and MCS-12 score by 0.48 and 0.57 respectively. Similarly, a two-s.d. higher BF% (about 22.3%) was associated with approximately 0.42 points lower GHQ (p-value: 0.008).The association between BF% and MCS-12 was not-significant. The WC regression coefficients were also negative and significant (p-value<0.001); a two-s.d. higher WC (about 28.3 cm) was related to the most prominent reduction in GHQ-12 and MCS-12 scores (about 0.74 and 0.97 lower, respectively).

**Table 2:** Association of adiposity measures with mental health functioning in UKHLS (waves 2–3, 5/2010-7/2013): Basic model^a^.

**GHQ-12 (inverted)**		
	Coefficient	95% CI
ΒΜΙ measures		
BMI (kg/m^2^)	-0.046***	-0.071, -0.021
Obese (BMI)	-0.528***	-0.801, -0.255
BF% measures		
BF%	-0.019**	-0.033, -0.005
Obese (BF%)	-0.456***	-0.718, -0.195
WC measures		
WC (cm)	-0.026***	-0.036, -0.016
Abdominal obese	-0.552***	-0.794, -0.310
**MCS-12**		
	Coefficient	95% CI
ΒΜΙ measures		
BMI (kg/m2)	-0.055*	-0.097, -0.013
Obese (BMI)	-0.646**	-1.096, -0.195
BF% measures		
BF%	-0.023	-0.047, 0.002
Obese (BF%)	-0.764**	-1.218, -0.310
WC measures		
WC (cm)	-0.034***	-0.051, -0.017
Abdominal obese	-0.608**	-1.027, -0.188
Sample size	11,257	

Abbreviations: BF%, percentage body fat; BMI, body mass index; CI, confidence interval; GHQ-12, 12-item General Health Questionnaire; MCS-12, Mental Component Summary of the 12-item Short Form Health Survey; WC, waist circumference

*P<0.05

**P<0.01

***P<0.001

^a^ Basic models accounted for demographic characteristics, regional/urbanization dummies and the time gap variable. Separate regression models were estimated for each adiposity measure (linear or dichotomous) with (inverted) GHQ-12 and MCS-12. All estimations accounted for sample weights and complex survey design.

Obesity, defined using BMI, was associated with lower GHQ-12 (β = -0.528, p-value<0.001) and MCS-12 scores (β = -0.646, p-value 0.005). Significant negative associations were observed between BF%-based obesity and both measures of mental health functioning; MCS-12 (β = -0.456, p-value<0.001) and GHQ-12 (β = -0.764, p-value = 0.002). On average, people with abdominal obesity had lower GHQ-12 and MCS-12 scores by 0.552 (p-value<0.001) and 0.608 (p-value = 0.005) respectively, compared with their non-obese counterparts.

### Heterogeneity by Age

**Fig 1:**
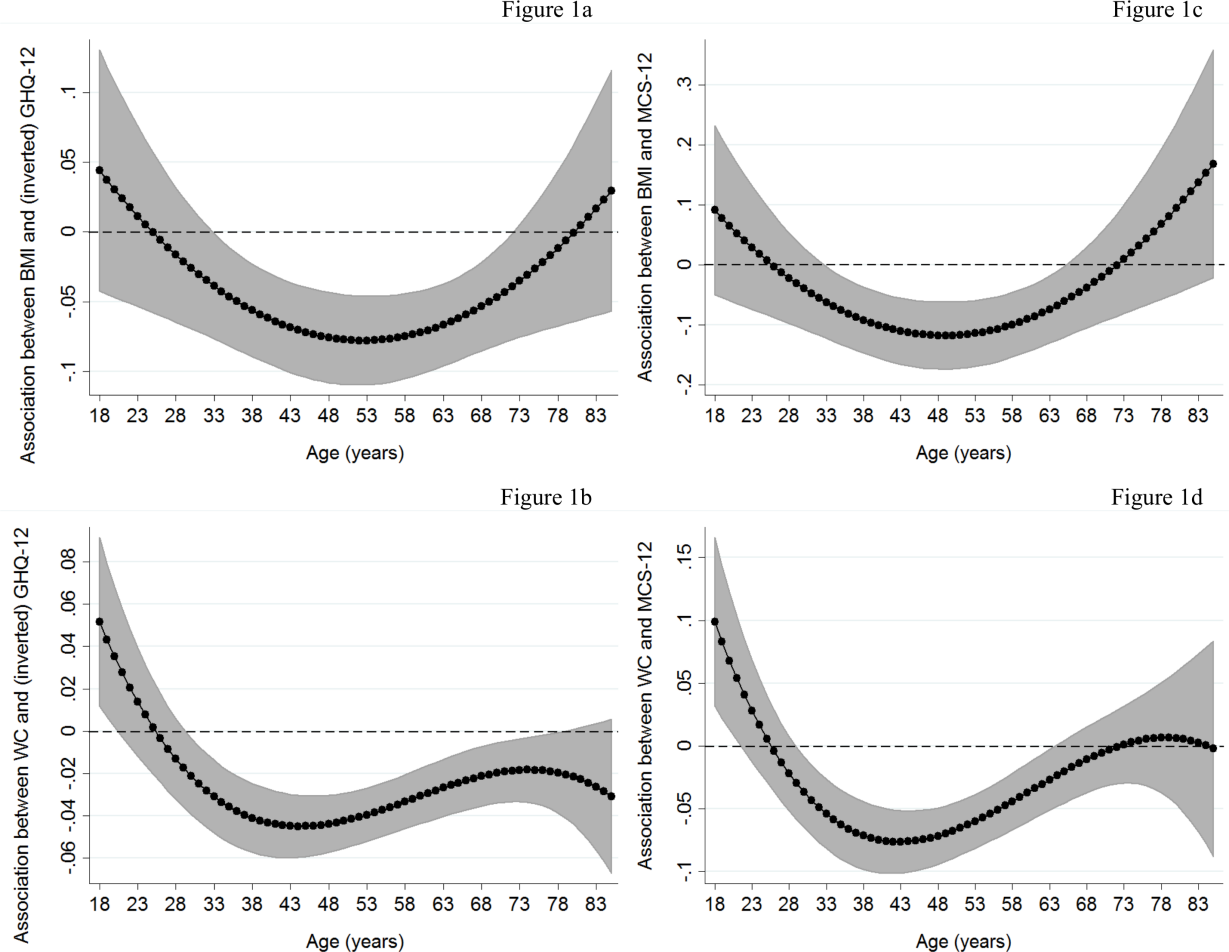
Association of continuous adiposity measures with mental health functioning by age: Basic model. The graphs present associations of adiposity measures with mental health functioning by age estimated by augmenting basic models (Table 2) with polynomial interactions of age with adiposity measures (linear and quadratic interactions in case of BMI; linear, quadratic and cubic interactions in case of WC). These associations were obtained by linear combinations of the main adiposity coefficients and the age-adiposity interactions across different ages (solid lines; shaded area: 95% confidence intervals).

Fig 1A and 1C (Fig 1) show the association of BMI with mental health functioning at different ages (solid lines; shaded area: 95%CI), obtained by including BMI-age polynomial interactions in the previous models (Table 2). The association of BMI with MCS-12 and GHQ-12 scores became negative and significant (zero lies outside the 95%CI) at around the age of 33 after which increased (became more negative) to the age of 50s (53 and 50 for GHQ-12 and MCS-12, respectively), where it peaked. The association then reduced until ages 72 for GHQ-12 and 66 for MCS-12, where it became non-significant at the 5% level.

Heterogeneous associations by age were also obtained for WC (Fig 1B and 1D). The negative association between WC and mental health functioning emerged at around the age of 30, peaked at 44, continued to decrease (less negative) and became non-significant at older ages (after 79 and 65 for GHQ-12 and MCS-12, respectively). Unlike BMI, a positive but diminishing WC-mental health functioning associations was observed in early young adulthood. There were no significant BF%-age interactions (at the 10% level) indicating no-heterogeneous age patterns.

**Fig 2:**
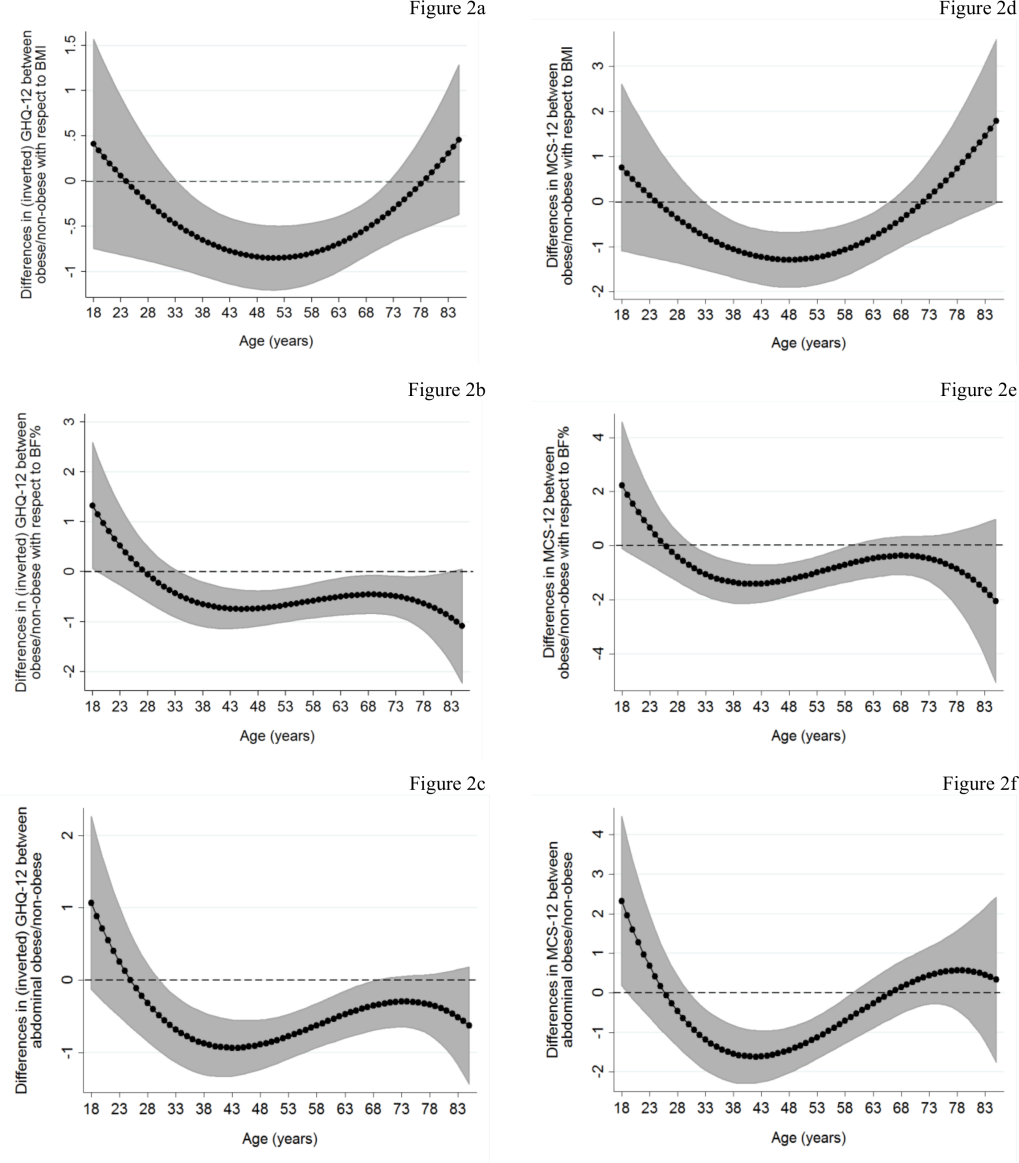
Association of dichotomous obesity measures with mental health functioning by age: Basic model. The graphs present predicted differences in mental health functioning between obese/non-obese by age estimated by augmenting basic models (Table 2) with polynomial interactions of age with obesity measures (linear and quadratic interactions in case of BMI-obesity measures; linear, quadratic and cubic interactions in case of BF%-obesity and abdominal obesity). These results were obtained by linear combinations of the main obesity coefficients and the age-obesity interactions across different ages (solid lines; shaded area: 95% confidence intervals).

Graphs for BMI-obesity (Fig 2A and 2D) reveal similar patterns to those for continuous BMI. For BF%-based obesity measures, people defined as obese experienced significantly lower GHQ-12 scores, compared to the non-obese, from the age of 35 (Fig 2B). These differences flattened at the age of 45 and smoothly decreased afterwards (becoming non-significant at the very old ages). The corresponding MCS-12 differences (Fig 2E) emerged and peaked at similar ages (31 and 41, respectively), however became non-significant after the age of 60. Age patterns obtained with abdominal obesity (Fig 2C and 2F) were similar to those from continuous WC measures (Fig 1B and 1D) with the exception that, unlike the latter, limited evidence in favor of the “jolly fat” hypothesis for the early young was observed.

### Examining the Role of Socio-Economic Factors, Marital Status, Smoking and Physical Health Conditions

**Fig 3:**
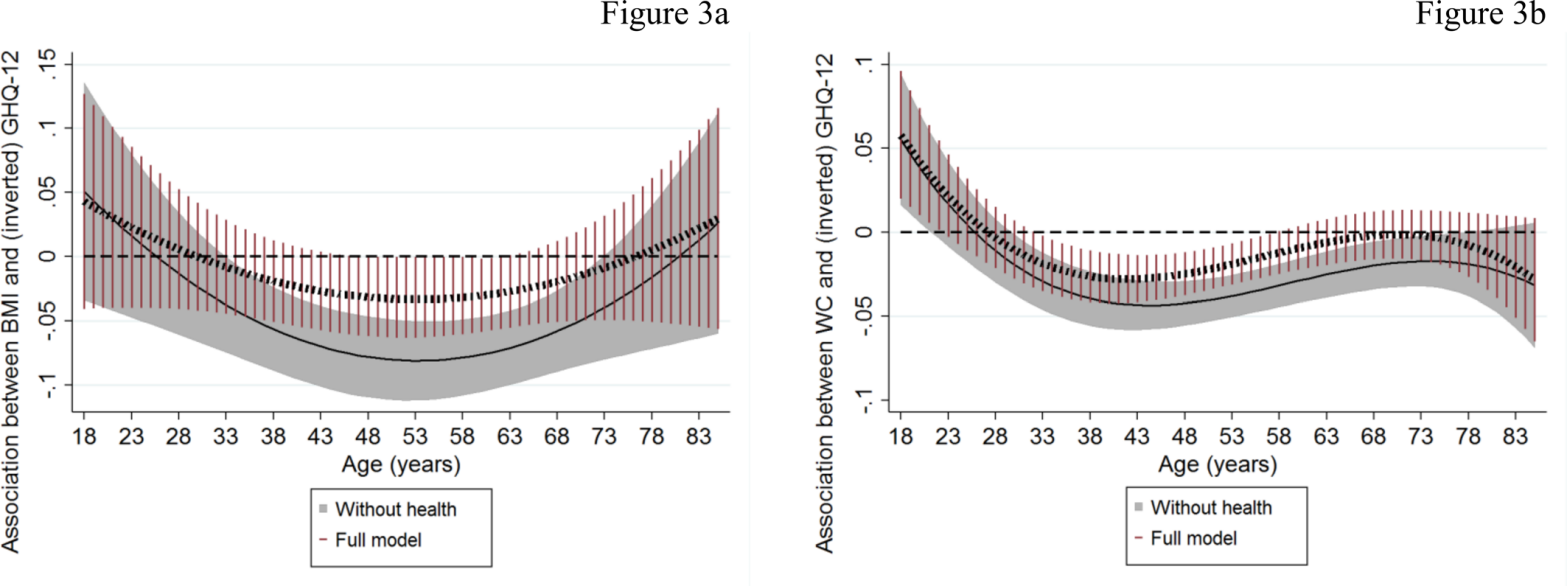
Association of continuous adiposity measures with GHQ-12 by age: the dominant role of physical health. The graphs present associations of adiposity measures with mental health functioning by age estimated when previous models (Fig 1) were further adjusted for (a) socio-economic characteristics, marital status and smoking (“without health”; solid lines with shaded area representing the 95%CI) and (b) for all the previous including the full set of physical health conditions (“full model”; dashed lines with spike plots representing the 95%CI). These associations were obtained by linear combinations of the main adiposity coefficients and the age-adiposity interactions (linear and quadratic interactions in case of BMI; linear, quadratic and cubic interactions in case of WC) across different ages.

**Fig 4:**
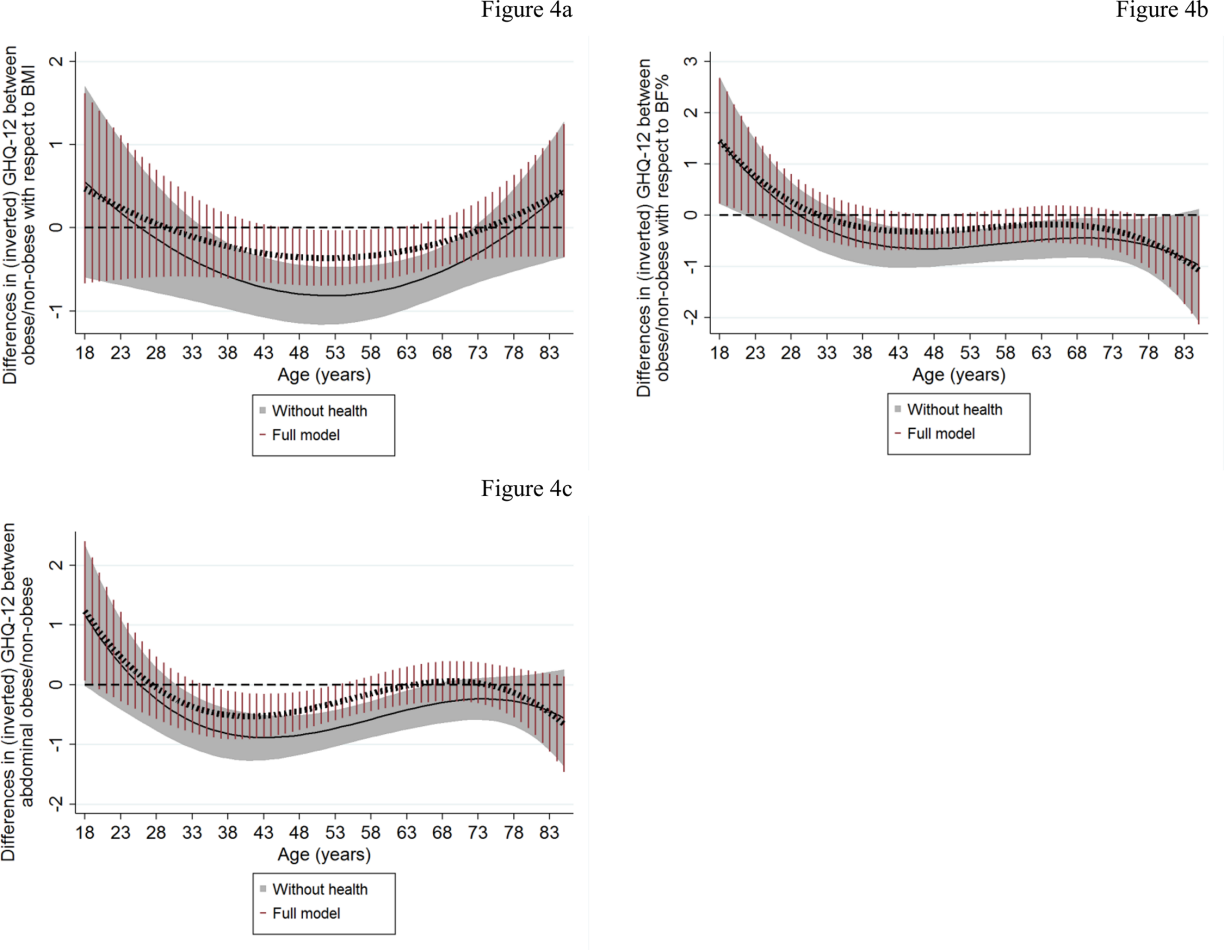
Association of dichotomous obesity measures with GHQ-12 by age: the dominant role of physical health. The graphs present predicted differences in mental health functioning between obese/non-obese by age estimated when previous models (Fig 2) were further adjusted for (a) socio-economic characteristics, marital status and smoking (“without health”; solid lines with shaded area representing the 95%CI) and (b) for all the previous including the full set of physical health conditions (“full model”; dashed lines with spike plots representing the 95%CI). These associations were obtained by linear combinations of the main obesity coefficients and the age-obesity interactions (linear and quadratic interactions in case of BMI-obesity; linear, quadratic and cubic interactions in case of BF%-obesity and abdominal obesity) across different ages.

Figs 3 and 4 present the association between adiposity measures and GHQ-12 by age with the previous models (Figs 1 and 2) further augmented (a) for the full set of covariates except health conditions (“without health” models; shaded area: 95%CI) and (b) for all the covariates including physical health conditions (“full model”; spike plots: 95%CI). Corresponding graphs for MCS-12 are presented as S1 Fig.

In general, the patterns of the association of adiposity measures (continuous and dichotomous) with mental health functioning by age did not change between the “without health” models (Figs 3 and 4 and S1 Fig) and the base models (Figs 1 and 2). However, adjusting for all physical health conditions (“full model”) generally attenuated the negative associations for all measures, except central adiposity, across the whole age range. The association between central adiposity measures (WC and abdominal obesity) and mental health functioning remained significant (although reduced in magnitude) in those aged between mid-30s and 50s (Figs 3B and 4C and S1 Fig). At the age of 40, where the observed associations peaked (“full models”), a one-s.d. higher WC (about 14.2 cm) was related with a lower score of 0.38 and 0.72 in GHQ-12 and MCS-12 (p-value<0.001) respectively. For example, this is approximately equivalent to the effect size of household income in case of GHQ-12 or with roughly one third of the difference in mental health functioning between current/never smokers for both GHQ-12 and MCS-12 (data not shown). For abdominal obesity (Fig 4C and S1 Fig), the peak predicted differences between obese and non-obese respondents were 0.532 GHQ-12 and 1.032 MCS-12 (p-value<0.001) at similar ages (around the age of 40).

Results from the “full model” also revealed that accounting for health conditions strengthened the positive associations of BMI- and abdominal-based obesity measures with MCS-12 in older ages (S1 Fig). There was also suggestive evidence supporting a positive association of WC and obesity-BF% with mental health functioning in emerging-adulthood (Figs 3B and 4B and S1 Fig).

To explore the role of physical health further, we also investigated the effect of specific condition groups on the association between adiposity measures and GHQ-12 (MCS-12). Tables 3 and 4 for GHQ-12 (S1 and S2 Tables for MCS-12) show the percentage reduction in the adiposity-mental health functioning associations (obtained from the linear combination of the main adiposity coefficients and the age-adiposity interactions across different ages) between the “without health” models and models with each group of physical conditions added separately. These results are presented for the age interval at which the attenuating role of physical health was evident (broadly mid-30s to 60s; Figs 3 and 4 and S1 Fig). In essence, cardiovascular diseases followed by arthritis and endocrine diseases resulted in the largest percentage reductions in the association between adiposity and mental health functioning. The percentage reduction tended to increase with age according to the majority of our models (Tables 3 and 4 and S1 and S2 Tables). However, no single condition fully attenuated the associations in the way that including them all in the model simultaneously did (Figs 3 and 4 and S1 Fig).

**Table 3:** Contribution of each physical health condition to the association between continuous adiposity measures and GHQ-12 at specific ages.

	Age (years)						
	35	40	45	50	55	60	65
**BMI**							
*% association explained by*^*a*^							
(1) Arthritis	21.5	21.2	21.3	21.5	22.0	22.8	24.1
(2) Cardiovascular diseases	33.3	30.2	28.9	28.4	28.4	28.9	30.0
(3) Endocrine diseases	16.7	15.2	14.6	14.4	14.5	14.9	15.8
(4) Respiratory diseases	14.0	9.9	7.9	6.8	6.1	5.7	5.6
(5) Other diseases	3.1	2.4	2.1	2.0	2.0	2.1	2.3
**WC**							
*% association explained by*^a^							
(1) Arthritis	9.9	10.3	11.6	13.7	16.6	20.3	24.4
(2) Cardiovascular diseases	16.9	17.5	19.4	22.3	26.3	31.4	36.5
(3) Endocrine diseases	8.6	8.4	9.2	10.7	13.0	16.2	19.8
(4) Respiratory diseases	5.7	5.2	5.5	6.4	8.1	10.4	13.3
(5) Other diseases	1.0	2.6	3.3	4.3	5.6	7.1	8.3

Abbreviations: BMI, body mass index; GHQ-12, 12-item General Health Questionnaire; WC, waist circumference

^a^ Percentage reduction in the association between continuous adiposity measures and GHQ-12 (obtained from the linear combination of the main adiposity coefficients and the age-adiposity interactions varying age from 35 to 65 years old) between the “without health” models (Fig 3) and models with each group of physical conditions added separately.

**Table 4:** Contribution of each physical health condition to the association between obesity and GHQ-12 at specific ages.

		Age (years)					
	35	40	45	50	55	60	65
**Obese (BMI)**							
*% association explained by*^a^							
(1) Arthritis	17.6	18.7	19.3	20.0	20.8	21.1	24.1
(2) Cardiovascular diseases	33.7	30.0	28.5	28.0	28.0	28.8	30.6
(3) Endocrine diseases	16.0	14.9	14.6	14.5	14.8	15.4	16.5
(4) Respiratory diseases	12.2	7.7	5.7	4.7	4.3	4.4	5.2
(5) Other diseases	3.9	3.1	2.6	2.2	1.9	1.5	0.9
**Obese (BF%)**							
*% association explained by*^a^							
(1) Arthritis	12.8	12.8	14.6	17.3	20.7	24.0	25.5
(2) Cardiovascular diseases	33.6	26.8	25.4	25.6	26.3	26.5	24.5
(3) Endocrine diseases	8.7	12.9	12.6	13.1	14.0	14.6	13.9
(4) Respiratory diseases	10.2	8.0	7.5	8.0	9.1	10.4	11.2
(5) Other diseases	7.6	5.1	4.2	3.7	3.2	2.6	1.7
**Abdominal obese**							
*% association explained by*^a^							
(1) Arthritis	8.4	10.2	12.8	16.5	21.7	28.9	37.9
(2) Cardiovascular diseases	19.7	20.0	21.9	25.3	30.4	37.8	47.3
(3) Endocrine diseases	8.8	8.8	10.0	12.0	15.0	19.5	25.4
(4) Respiratory diseases	9.5	7.6	7.3	7.8	9.0	11.1	13.9
(5) Other diseases	2.0	2.0	2.3	2.8	3.6	4.7	6.3

Abbreviations: BF%, percentage body fat; BMI, body mass index; GHQ-12, 12-item General Health Questionnaire; WC, waist circumference

^a^ Percentage reduction in the association between obesity and GHQ-12 (obtained from the linear combination of the main adiposity coefficients and the age-adiposity interactions varying age from 35 to 65 years old) between the “without health” models (Fig 4) and models with each group of physical conditions added separately.

## Discussion

Using data from a large nationally representative sample of people across the adult age span (UKHLS), this study found that the association between adiposity (measured by BMI, WC and BF% using continuous or dichotomous obesity indicators) and mental health functioning (assessed by both psychological distress and MHRQoL) followed heterogeneous patterns by age. In general, we found negative adiposity-mental health functioning associations that emerged in people aged in their early 30s, increased up to mid-40s (all WC and obesity-BF% measures) or early 50s (all BMI measures)—where they peaked—and then decreased. No age trajectories were observed for continuous BF%.

However, accounting for physical health conditions significantly reduces the negative adiposity-mental health functioning associations. These associations were fully accounted for in case of BMI- and BF%-based adiposity measures, but remained apparent for central adiposity measures in those aged between mid-30s to 50 years. Examining physical health conditions separately, suggested that cardiovascular, followed by arthritis and endocrine, conditions resulted in the largest attenuations.

Allowing for flexibility in the association between adiposity and mental health functioning by age, this study demonstrated strong age-specific patterns which are often masked in previous studies [14, 15]. Our evidence supports and extends previous literature by employing alternative measures of adiposity and examining these associations across different ages. Previous studies suggest that abdominal obesity is association with depression or depressive symptoms [4]; our findings extend this evidence to psychological distress and MHRQoL.

In relation to age heterogeneity in the association between adiposity and mental health functioning, our findings are broadly in line with the few studies available that suggest negative associations in younger rather than older individuals [11, 16]. A number of factors may explain these age patterns. For example, interpersonal discrimination or social isolation potentially experienced by people who are obese [14] may be greater at younger than older ages [41]. Moreover, increased adiposity may be considered normative in older age groups [42] and, thus, may be less associated with poor mental health.

The dominant role of underlying physical health in (partially or mainly) explaining the association between adiposity and mental health functioning is generally in accordance with previous studies [43, 44]. The fact that cardiovascular diseases exerted the greatest attenuations may reflect their high prevalence and their strong correlation with both adiposity and mental health [17, 20, 45]. Shared disease mechanisms, such as overlap in the risk factors, physiological changes regarding nervous system activation and cardiac rhythm disturbances as well as stress [46], and common symptoms [47] may explain the strong association between mental health and cardiovascular disorders. However, it is difficult to determine causality and direction of associations in our analyses. For example, cardiovascular risk factors are strongly associated with mental health and adiposity with a number of possible bi-directional pathways. The attenuating role of physical conditions increased with age in most of our models, which may reflect the increase in prevalence at older ages.

The continued association of WC and abdominal obesity with mental health functioning, after controlling for physical health, confirms previous stronger associations in case of abdominal obesity rather than other adiposity measures [22]. This suggests other factors may operate for WC, compared to the other adiposity measures. For example, central adiposity, rather than generalized obesity, may better reflect psychosocial adversity [48]; this is of particular importance assuming that psychosocial factors among obese individuals may be involved in the psychopathology of mental health [9]. Similarly, abdominal obesity may be important as it plays a significant role in poor self-body image [49].

We found no support for the moderating role of gender on these associations in accordance with some [15], but not all [14] existing research. These disparities may relate to differences in the age ranges, populations and mental health measures employed across studies. For instance, although a recent UK study suggested there were gender differences [50], it was limited to a narrow age range and examined more severe mental health problems compared to our study.

Our evidence may have important implications that need to be highlighted. The inter-relatedness of obesity, chronic diseases and mental health demonstrated in this study, suggests that prevention policies aimed at reducing obesity and cardiovascular disease may have beneficial effects on mental health. Treatment of any of these problems in isolation may be less effective than addressing them in combination. Finally, differences in our results across adiposity measures reinforce the importance of considering alternative adiposity measures to better understand obesity and its causes and consequences.

This paper is the first to provide a thorough examination of the association between different adiposity measures and mental health functioning allowing for flexible age gradients in the associations. However, there are a number of potential limitations. Firstly, analyses are almost cross sectional in nature (since the gap between adiposity measurements and mental health functioning is fairly narrow, i.e. about 7 months on average) and, thus, we are not able to make firm assertions about causality. Secondly, although this paper may highlight the importance of considering a wide age range in the case of cross-sectional analysis of the adiposity-mental health functioning association, we cannot disentangle age and cohort effects. Further longitudinal research is needed to elucidate these issues. Thirdly, our study is predominantly composed of White/European participants and therefore we cannot generalize to non-White groups. However, studies suggesting ethnic group differences largely emanate from the US [11] and may not apply to the UK ethnic minority groups. Fourthly, our measures of chronic conditions are based on self-reported, rather than clinical, data which may lead to measurement error. Finally, there is a high level of attrition and item missingness in our analysis; the former may be accounted for by sample weights which are employed, while differences between the analytical and full sample suggests limited impact of the latter. The possibility of health survivor effects distorting the associations at older ages may also be a concern.

In summary, we found that the negative associations between adiposity measures and mental health functioning are characterized by strong age heterogeneity, which may explain mixed findings in the literature. These associations remained unaffected after adjusting for several potential confounders except underline physical health. After adjusting for our set of physical health conditions, the negative adiposity-mental health functioning associations were limited to central adiposity measures in the mid-30s to 50s age group. Cardiovascular, followed by arthritis and endocrine, diseases played the greatest role in explaining associations. Suggestive results about positive adiposity-mental health functioning associations in young adulthood and older ages are also evident.

## Supporting Information

S1 Fig Association of adiposity measures with MCS-12 by age: the dominant role of physical health. The graphs present the corresponding results to those in Figs 3 and 4 for the case of MCS-12.
(PDF)

S2 Fig Flow chart describing analytical sample. (PDF)

S1 Table Contribution of each physical health condition to the association between continuous adiposity measures and MCS-12 at specific ages. (PDF)

S2 Table Contribution of each physical health condition to the association between obesity and MCS-12 at specific ages. (PDF)

S3 Table STROBE Statement—Checklist of items that should be included in reports of cross-sectional studies. (PDF)
