# Supplementary material for: Association of Adiposity and Mental Health Functioning across the Lifespan: Findings from Understanding Society (The UK Household Longitudinal Study)
Source: PLoS One. 2016 Feb 5;11(2):e0148561. doi: 10.1371/journal.pone.0148561 (PMC4744034; doi:10.1371/journal.pone.0148561)

S1 Fig. Age variations in the association of adiposity measures with MCS-12: the dominant role of physical health.

Linear adiposity measures **Obesity measures**

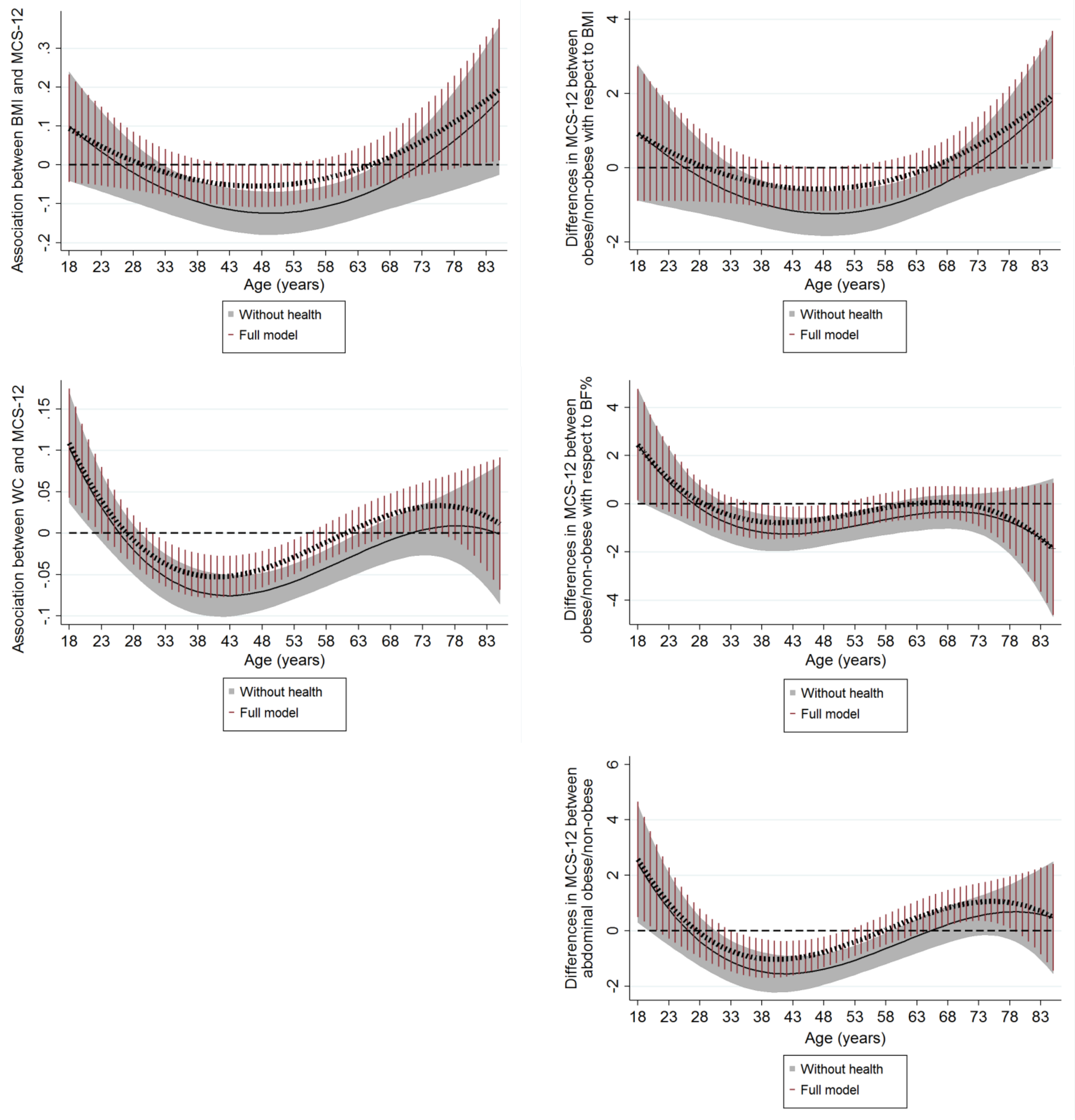

Supplement: S1 Fig — The graphs present the corresponding results to those in Figs 3 and 4 for the case of MCS-12. (PDF) [file pone.0148561.s001.pdf]
