# Supplementary figures and images for: Association of Adiposity and Mental Health Functioning across the Lifespan: Findings from Understanding Society (The UK Household Longitudinal Study)
Source: PLoS One. 2016 Feb 5;11(2):e0148561. doi: 10.1371/journal.pone.0148561 (PMC4744034; doi:10.1371/journal.pone.0148561)

**S2 Fig. Flow chart describing analytical sample.**

---

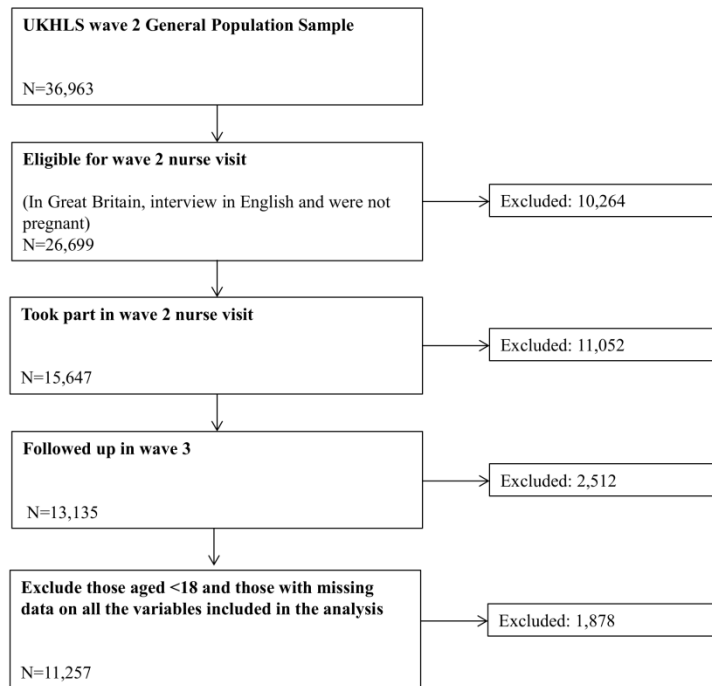

Supplement: S2 Fig — (PDF) [file pone.0148561.s002.pdf]
