# Supplementary material for: Association of Adiposity and Mental Health Functioning across the Lifespan: Findings from Understanding Society (The UK Household Longitudinal Study)
Source: PLoS One. 2016 Feb 5;11(2):e0148561. doi: 10.1371/journal.pone.0148561 (PMC4744034; doi:10.1371/journal.pone.0148561)
Supplement: S1 Table — (PDF) [file pone.0148561.s003.pdf]

S1 Table. Contribution of each physical health condition to the association between continuous adiposity measures and MCS-12 at specific ages.

	Age (years)					
	**35**	**40**	**45**	**50**	**55**	**60**
**BMI**						
% association explained by [a]						
(1) Arthritis	13.1	13.2	13.6	14.5	16.0	18.9
(2) Cardiovascular diseases	33.0	31.1	30.5	31.2	33.2	37.5
(3) Endocrine diseases	10.4	10.7	11.8	13.8	17.1	23.2
(4) Respiratory diseases	14.9	10.7	8.8	7.9	7.7	8.1
(5) Other diseases	3.2	3.8	3.9	4.3	4.9	6.2
**WC**						
% association explained by [a]						
(1) Arthritis	5.7	8.2	9.9	12.6	13.7	17.1
(2) Cardiovascular diseases	16.4	17.1	19.4	23.4	30.3	43.5
(3) Endocrine diseases	4.8	5.4	7.1	10.1	15.6	27.0
(4) Respiratory diseases	5.9	5.3	5.8	7.2	10.0	15.8
(5) Other diseases	1.0	1.0	1.3	1.9	3.1	5.8

Abbreviations: BMI, body mass index; MCS-12, Mental Component Summary of the 12-item Short Form Health Survey; WC, waist circumference

[a] Percentage reduction in the association between continuous adiposity measures and MCS-12 (obtained from the linear combination of the main adiposity coefficients and the age-adiposity interactions varying age from 35 to 60 years old) between the “without health” models (S1 Fig) and models with each group of physical conditions added separately.
