# Supplementary material for: Association of Adiposity and Mental Health Functioning across the Lifespan: Findings from Understanding Society (The UK Household Longitudinal Study)
Source: PLoS One. 2016 Feb 5;11(2):e0148561. doi: 10.1371/journal.pone.0148561 (PMC4744034; doi:10.1371/journal.pone.0148561)
Supplement: S3 Table — (PDF) [file pone.0148561.s005.pdf]

S3 Table. STROBE Statement—Checklist of items that should be included in reports of cross-sectional studies.

	Item No	Recommendation	Reported
**Title and abstract**	1	(*a*) Indicate the study's design with a commonly used term in the title or the abstract	Abstract
		(*b*) Provide in the abstract an informative and balanced summary of what was done and what was found	Abstract
**Introduction**			
Background/rationale	2	Explain the scientific background and rationale for the investigation being reported	Introduction
Objectives	3	State specific objectives, including any prespecified hypotheses	Introduction
**Methods**			
Study design	4	Present key elements of study design early in the paper	Methods (sub-section: sample)
Setting	5	Describe the setting, locations, and relevant dates, including periods of recruitment, exposure, follow-up, and data collection	Methods (sub-section: sample)
Participants	6	(*a*) Give the eligibility criteria, and the sources and methods of selection of participants	Methods (sub-section: sample)
Variables	7	Clearly define all outcomes, exposures, predictors, potential confounders, and effect modifiers. Give diagnostic criteria, if applicable	Methods (sub-section: measures)
Data sources/ measurement	8	For each variable of interest, give sources of data and details of methods of assessment (measurement). Describe comparability of assessment methods if there is more than one group	Methods (sub-section: measures)
Bias	9	Describe any efforts to address potential sources of bias	Methods (sub-section: data analysis) and Discussion
Study size	10	Explain how the study size was arrived at	Methods (sub-section: sample)
Quantitative variables	11	Explain how quantitative variables were handled in the analyses. If applicable, describe which groupings were chosen and why	Methods
Statistical methods	12	(*a*) Describe all statistical methods, including those used to control for confounding	Methods (sub-section: data analysis)
		(*b*) Describe any methods used to examine subgroups and interactions	Methods (sub-section: data analysis)
		(*c*) Explain how missing data were addressed	Methods (sub-section: data analysis) and discussion
		(*d*) If applicable, describe analytical methods taking account of sampling strategy	Methods (sub-section: data analysis)
		(*e*) Describe any sensitivity analyses	n/a
**Results**			
Participants	13	(a) Report numbers of individuals at each stage of study—eg numbers potentially eligible, examined for eligibility, confirmed eligible, included in the study, completing follow-up, and analysed	Results
		(b) Give reasons for non-participation at each stage	Methods (sub-section: sample) and Results
		(c) Consider use of a flow diagram	S2 Fig, supplementary information
Descriptive data	14	(a) Give characteristics of study participants (eg demographic, clinical, social) and information on exposures and potential confounders	Results
		(b) Indicate number of participants with missing data for each variable of interest	Results
Outcome data	15	Report numbers of outcome events or summary measures	Table 1
Main results	16	(*a*) Give unadjusted estimates and, if applicable, confounder-adjusted estimates and their precision (eg, 95% confidence interval). Make clear which confounders were adjusted for and why they were included	Results, Figures 3 and 4
		(*b*) Report category boundaries when continuous variables were categorized	Methods (sub-section: measures)
		(*c*) If relevant, consider translating estimates of relative risk into absolute risk for a meaningful time period	n/a
Other analyses	17	Report other analyses done—eg analyses of subgroups and interactions, and sensitivity analyses	Methods and results
**Discussion**			
Key results	18	Summarise key results with reference to study objectives	Discussion
Limitations	19	Discuss limitations of the study, taking into account sources of potential bias or imprecision. Discuss both direction and magnitude of any potential bias	Discussion
Interpretation	20	Give a cautious overall interpretation of results considering objectives, limitations, multiplicity of analyses, results from similar studies, and other relevant evidence	Discussion
Generalisability	21	Discuss the generalisability (external validity) of the study results	Discussion
**Other information**			
Funding	22	Give the source of funding and the role of the funders for the present study and, if applicable, for the original study on which the present article is based	Cover letter
